# Development and validation of a nomogram based on lymphocyte subsets to distinguish bipolar depression from major depressive disorder

**DOI:** 10.3389/fpsyt.2022.1017888

**Published:** 2022-10-06

**Authors:** Liming Su, Yibing Shuai, Shaoqi Mou, Yue Shen, Xinhua Shen, Zhongxia Shen, Xiaomei Zhang

**Affiliations:** ^1^Department of Neurosis and Psychosomatic Diseases, Huzhou Third Municipal Hospital, The Affiliated Hospital of Huzhou University, Huzhou, China; ^2^Department of Psychiatry, Wenzhou Medical University, Wenzhou, China

**Keywords:** bipolar depression (BD), major depressive disorder (MDD), lymphocyte subsets, differential diagnosis, nomogram

## Abstract

**Objective:**

Bipolar depression (BD) and major depressive disorder (MDD) are both common affective disorders. The common depression episodes make it difficult to distinguish between them, even for experienced clinicians. Failure to properly diagnose them in a timely manner leads to inappropriate treatment strategies. Therefore, it is important to distinguish between BD and MDD. The aim of this study was to develop and validate a nomogram model that distinguishes BD from MDD based on the characteristics of lymphocyte subsets.

**Materials and methods:**

A prospective cross-sectional study was performed. Blood samples were obtained from participants who met the inclusion criteria. The least absolute shrinkage and selection operator (LASSO) regression model was used for factor selection. A differential diagnosis nomogram for BD and MDD was developed using multivariable logistic regression and the area under the curve (AUC) with 95% confidence interval (CI) was calculated, as well as the internal validation using a bootstrap algorithm with 1,000 repetitions. Calibration curve and decision curve analysis (DCA) were used to evaluate the calibration and clinical utility of the nomogram, respectively.

**Results:**

A total of 166 participants who were diagnosed with BD (83 cases) or MDD (83 cases), as well as 101 healthy controls (HCs) between June 2018 and January 2022 were enrolled in this study. CD19^+^ B cells, CD3^+^ T cells, CD3^–^CD16/56^+^ NK cells, and total lymphocyte counts were strong predictors of the diagnosis of BD and MDD and were included in the differential diagnosis nomogram. The AUC of the nomogram and internal validation were 0.922 (95%; CI, 0.879–0.965), and 0.911 (95% CI, 0.838–0.844), respectively. The calibration curve used to discriminate BD from MDD showed optimal agreement between the nomogram and the actual diagnosis. The results of DCA showed that the net clinical benefit was significant.

**Conclusion:**

This is an easy-to-use, repeatable, and economical nomogram for differential diagnosis that can help clinicians in the individual diagnosis of BD and MDD patients, reduce the risk of misdiagnosis, facilitate the formulation of appropriate treatment strategies and intervention plans.

## Introduction

Bipolar disorder (BD) and major depressive disorder (MDD) are two significant spectra of mental disorders characterized by clinical symptoms triggered by dysfunction in the emotional, cognitive and behavioral, and somatic domains (1). It has been reported that the lifetime prevalence of BD and MDD is 5 and 16.2% (2, 3), respectively, and the misdiagnosis of BD as MDD was as high as 40% (4), so the over-diagnosis of MDD is partly caused by the high rate of misdiagnosis of BD; which can be modified by adequate recognition and accuracy in diagnosing MDD from BD (5). In general, patients with BD who have a history of evident mania can be separated from those with MDD (6). However, most patients with BD experience milder or atypical forms of (hypo) mania, which are frequently overlooked by patients and clinicians (7). In addition, Perlis et al. found that it takes an average of 8 years time from the first attack of patients with BD to the proper diagnosis and treatment (8). Unfortunately, the accurate diagnosis of the two diseases can be challenging even for experienced physicians. During depressive episodes, there are overlapping clinical presentations of BD and MDD (9, 10), in which anxiety symptoms are the most commonly comorbidity symptoms (11) and the severity of current anxiety symptoms are strongly associated with the subsequent persistence of depressive symptoms (12). These cause the misdiagnosis of each other. It is noteworthy that the treatment strategies for these two diseases are quite different. In clinical practice, patients with BD have been prescribed with mood stabilizers (such as lithium salts, antiepileptics, and antipsychotics), whereas patients with MDD are prescribed with antidepressants only (13). Additionally, there is considerable debate regarding the effectiveness of antidepressants for BD (14). Given the high rate of misdiagnosis of BD and the differences in treatment strategies between BD and MDD, it is essential to make a correct diagnosis of BD and MDD. Otherwise, it will lead to the use of inappropriate drug treatment, with subsequent adverse outcomes and poor prognosis (e.g., prolonged morbidity, suicide, mania, and disability) (15–17), which severely impairs the quality of life of patients, and further increases the healthcare burden.

Currently, there is no gold standard for diagnosing BD and MDD. The existing diagnostic consensus points out that a comprehensive judgment should be combined with socio-demographics, clinical features, biological markers, brain electrophysiology, neuroimaging examinations, and treatment conditions (9). Nevertheless, there are still many issues to be investigated in the research and practice of BD and MDD identification. In recent years, it has been confirmed that the immune response plays a vital role in the pathogenesis of affective disorders (18, 19), especially lymphocyte subtypes consisting of Breg (B), Treg (T), and natural killer (NK) cells, have been proposed as inflammatory markers and supporting the inflammatory hypothesis underlying the etiopathogenesis of these conditions (19). Regarding the lymphocyte subtypes with the following characteristics and physiological functions: (1) B cells can be developed into plasma cells, which produce the antibodies important against foreign intruders, mainly represented by extracellular bacteria, and participate in the humoral immune response (20), and CD19^+^ is one of the important membrane antigens involved in B cell activation and proliferation, and is a common surface marker for all B cells (21). (2). T cells are antigen specific, generated in the thymus, can be identified based on “cluster of differentiation” (CD) proteins expressed on their surface and/or on the cytokines they produce and are capable of differentiating into T cytotoxic or T helper cells (19, 20). The former present antigens to B cells, while the latter inhibit the function of effector T cells, B cells, and the proliferation of lymphocytes (20). All CD3^+^ T cells express the typical T cell receptor, playing a role in antigen recognition (22). (3) NK cells are predominantly large granular lymphocytes (LGL), which express CD 16 and/or CD 56 surface antigens, have anti-tumor, anti-infection, immunomodulatory, and hematopoietic effects, and are essential lymphocytes in the natural immune system, and their killing effects are spontaneous and generally do not require the presence of antibodies or pre-sensitization (19).

Indeed, lymphocyte subtypes were performed by collecting blood samples and calculating the corresponding parameters, which has the advantage of being low-cost, reproducible, and easily available under simple laboratory conditions. Up to the present date, several studies have examined the usefulness of lymphocyte subsets as potential biomarkers of neuro-inflammatory response with BD and MDD (23–25). For instance, a study by Bauer et al. showed that when patients with BD or MDD experienced acute psychological stress, the circulation of lymphocytes dysregulated, including the number of lymphatic subsets, and the corresponding functional impairment (1). Breunis, et al. found that patients with symptomatic and remitted BD had higher levels of activated CD3^+^ T cells compared to healthy controls (HCs) (22) and the same results were obtained in the study of Barbosa et al. (26). However, 21 BD type I patients and 21 age- and sex-matched controls were recruited for a study by Barbosa et al. concluded that BD patients presented reduced proportions of CD3^+^ T cells (26). Denney et al. observed in 15 MDD patients a decreased number of CD3^+^ cells relative to HCs (27), but in a study of 20 patients with MDD, a slight increase in CD3^+^ cells was described, but did not reach a significant difference with HCs (28). Besides, Müller et al., evaluated 37 patients suffering from a subtype of endogenous depression (during the depression and during free intervals), and found significantly higher levels of CD3^+^ cells compared to HCs (29). Wu et al. reported that the distribution of T cells in BD patients was significantly different from that in MDD patients and patients with BD had significantly lower levels of cytotoxic T cells than patients with MDD (19, 25). The evidence from studies related to T-cell subsets in BD and MDD has not been clearly conclusive, indicating a gap in the field that deserves to be filled. Additionally, several studies have pointed to abnormal numbers and activity of B and NK cells in affective disorders. Mays et al. investigated whether the number or percentage of B cells reflected depressive states and noticed that depressed patients had significantly higher numbers of CD19^+^ B cells compared to HCs (30). A recent study that compared BD patients with HCs on distribution of B lymphocyte subsets yielded a similar result: patients with BD who were in remission and depressive episodes had a higher percentage of CD19^+^ B cells than HCs (31). The last but not least, Benschop et al. showed that acute psychological stress induces a transient increase in lymphocyte numbers, with NK cell counts being the most prominent (32); on the contrary, Patas et al. discovered that decreased NK cell counts were observed in patients with MDD (33, 34). Studies on exploration of NK cell levels in BD are lacking, and the differences in level of NK and B cell between BD and MDD are unclear. In light of the close relationship of immune response between BD and MDD, B and NK cells may play different roles, though the specific mechanism of this association remains to be fully determined. Taken together, these findings suggest that additional more investigation into differences in lymphocyte subsets between BD and MDD is necessary to reveal the relevant pathophysiological mechanisms and assist in clinical diagnosis.

Typically, because of the interplay of variables among lymphocyte subsets, focusing on the significance of a single factor may overlook the highly correlated factors from a clinical standpoint. Henceforth, to avoid these deficiencies, a clinical decision-making approach currently widely used in affective disorders – based on machine learning techniques, may help deal with complex factors, and show high predictive power (35, 36). Regretfully, it is rarely utilized to differentiate between BD and MDD disorders. Of these computational methods or models, the least absolute shrinkage and selection operator (LASSO) is one of the most widely utilized algorithms, which can comprehensively consider the synergistic effect among multiple influencing factors (37, 38), and select the most effective predicting factors from the available data set (35). Furthermore, a nomogram is an individualized and evidence-based predictive model that provides accurate information for decision-making objectively (39). As a result, applying machine learning techniques based on previous studies can provide a trustful methodological reference for this study to determine the predictors of BD and MDD.

The objectives of this study were as follows: (1) to explore the diagnostic value of lymphocyte-based subsets in distinguishing BD from MDD; (2) to develop and validate a differential diagnosis model to differentiate between BD and MDD with a base-machine learning technique, and was presented as a nomogram to improve the accuracy and usefulness of the predictive model. In all, in order to provide a simple and effective diagnostic tool for early differentiation of these two disorders and provide evidence support for follow-up clinical intervention.

## Materials and methods

### Study design

From June 2018 to January 2022, a prospective cross-sectional study was performed at Huzhou Third Municipal Hospital. This protocol was approved by the ethical review board of this institution and all participants provided signed informed consent before enrollment. In this study, assuming the nomogram could distinguish BD from MDD with a sensitivity and specificity of 90%, at least 158 participants (40).

Inclusion criteria were as follows: (1) age 18–65 years; (2) diagnosed with BD or MDD according to diagnostic and statistical manual of mental Disorders-IV (DSM-IV) criteria; (3) 17-item Hamilton rating scale for depression (HAM-D_17_) score ≥14. Exclusion criteria were as follows: (1) with immune system diseases (e.g., rheumatoid arthritis, systemic lupus erythematosus); (2) used anti-inflammatory or immunosuppressive drugs in the past year; (3) infectious diseases in the past 2 weeks; (4) comorbidity of other mental or nervous system diseases; (5) any clinically serious physical diseases (including chronic diseases such as hypertension, hyperlipidemia, and diabetes); (6) history of pregnancy and lactation; (7) blood dyscrasia, hepatic or renal failure, or obesity (body mass index (BMI) > 30 kg/m^2^); and (8) alcohol, drugs or other psychotropic substance use disorder.

Finally, 166 participants diagnosed with BD (*n* = 83) or MDD (*n* = 83) met the inclusion criteria. According to the match ratio of 1:1:1, 83 cases of HCs were required, and 101 cases of HCs were enrolled. This prediction model study was carried out in accordance with the Transparent reporting of a multivariable prediction model for individual prognosis or diagnosis (TRIPOD) checklist (41) (shown in Supplementary material 1).

### Information collection

Totally 29 factors, included clinical features [age, gender, height, weight, and BMI, onset age, illness duration, current episode duration, smoking, drinking, education level, employment status, marital status, family history of affective disorders, Hamilton rating scale for anxiety (HAMA) score, HAM-D_17_ score], and the characteristic of lymphocyte subsets [CD19^+^ B cell, CD19^+^ B cell (%), CD3^+^ total T cell, CD3^+^ total T cell (%), CD3^+^/CD4^+^ T-helper cell, CD3^+^/CD4^+^ T-helper cell (%), CD3^+^/CD8^+^ T-cytotoxic cell, CD3^+^/CD8^+^ T-cytotoxic cell (%), CD3^–^/CD16^+^56^+^ NK cell, CD3^–^/CD16^+^56^+^ NK cell (%), CD4^+^CD8^+^ (ratio), total lymphocyte count, and DN T (%)].

### Instruments for evaluating clinical symptoms

All participants underwent the structural clinical interview for DSM-IV disorders (SCID) by experienced psychiatrists and were finally diagnosed with BD or MDD according to DSM-IV. The Chinese version of the HAM-D_17_ (42) and HAMA (43) scales were used to determine the severity of depressed mood and anxiety symptoms in participants with BD and MDD, respectively. These were the most frequently used assessment tools in clinical practice.

### The procedure for collecting blood samples

Peripheral blood samples were collected after a 12 h overnight fast; approximately 2 mL of blood was drawn and placed in a blood sample tube containing EDTA anticoagulant. Blood samples were processed within 30 min of collection using BriCyte E6 flow cytometry (Mindray, Shenzhen, Guangdong, China) and performed in the Hangzhou Dian laboratory by technicians who were blinded to this study.

### The procedure for detecting lymphocyte subsets

According to the sample preparation instructions provided by the manufacturer, two flows of sample tubes should be taken, and they should be sequentially numbered with the test number; The procedure for using quality control materials (Beckman Coulte, Inc., Brea, CA, USA) is the same as the procedure for using standard sample tests. First, 10 μl of CD3/CD8/CD45/CD4 and CD3/CD16^+^CD56/CD45/CD19 antibodies (Mindray, Shenzhen) were injected into the numbered flow sampling tubes, respectively. After inverting and mixing the blood samples 6–8 times, 50 μl of blood samples were injected into the corresponding numbered tubes by the trans-addition technique, mixed well, and left at room temperature in the dark for 15 min. Then, 450 μl of red blood cell lysates (1:9 dilution with deionized water) were injected into each tube, mixed well, and left again at room temperature in the dark for 15 min. Finally, the tests were performed on a calibrated instrument.

### Statistical analysis

Statistical Package for the Social Sciences (SPSS) statistics software for windows (version 26.0) was used to make a descriptive statistical analysis for illustrating the clinical information and the characteristics of lymphocyte subsets in the HCs, BD, and MDD groups. Fisher’s exact test and Chi-square test were used to analyze proportional differences for categorical variables [*n* (%)], which were presented as frequency and percentage. Before applying statistical analysis to the continuous variables, the Shapiro–Wilk’s test was used to check the data normality; Continuous variables with a normal distribution were expressed as mean and standard deviation [Mean (S.D.)], and the univariate ANOVA was used for comparison between multiple groups and Bonferroni correction was used for pairwise comparison between groups and an independent *t*-test was performed to assess differences between BD and MDD groups in univariate analysis; Non-normal distribution continuous data was represented as median and interquartile range [Median (IQR)], the Kruskal–Wallis H test was used for comparison between multiple groups, and Nemenyi correction was used for pairwise comparison between groups, the Mann–Whitney U test was used to evaluate differences between BD and MDD groups in univariate analysis.

Rest analyses were conducted using R statistical software for windows (version 4.0.4). The LASSO regression algorithm with the “glmnet” package (44) was used to screen for factors that were significantly associated with BD versus MDD status and eliminate multicollinearity between factors. The performance of this classification algorithm is mainly determined by the parameter (lambda). As a result, 10-fold cross-validation (45) was adopted to select lambda with the minimum criteria. The optimal factors determined by LASSO analysis were further examined by univariate and multivariate logistic regression analysis, with the results demonstrated as odds ratio (OR) with 95% confidence interval (CI) and *P*-value. Subsequently, factors with statistically significant univariate (*P*-value < 0.05) were included multivariable logistic regression model after adjusting for age, sex, and BMI, and the backward stepwise selection was performed, with improvements in the goodness of fit measured by a decrease in the Akaike information criterion (AIC) (46). Following that, a differential diagnosis model was constructed based on the results of multivariate analysis.

Additionally, other R software packages that were used were (1) the “pROC” package for the receiver operating characteristic (ROC) curve graphics, which represented the area under the curve (AUC) and Harrell’s concordance index (C-index), as well as computed the optimal cut-off value; (2) the “rms” package was utilized to generate a developed nomogram and calibration curve in order to visualize and calibrate the differential diagnosis model; (3) the “generalhoslem” package was used to perform the Hosmer–Lemeshow test (47) for detected goodness-of-fit of the nomogram, the result was supported by a *P*-value > 0.05; and (4) the “rmda” package was applied to perform the decision curve analysis (DCA) to judge the clinical utility of the nomogram based on the net benefit and threshold probability (48). All these packages are available on the website.^1^ All *P*-value < 0.05 (two-sided) were considered statistically significant.

Ultimately, the accuracy of the nomogram was evaluated by internal validation through a bootstrap algorithm with 1,000 repetitions (49).

## Results

### Participants information

Gender, age, and lymphocyte subsets characteristics of the HCs (101 cases), BD (83 cases), and MDD (83 cases) groups were shown in Supplementary Table 1. Excluded sex-age differences and lymphocyte subsets were statistically different among the multiple comparisons. Further pairwise comparisons revealed that some lymphocyte subsets had statistically different comparisons of HCs and MDD group, HCs and BD group, and MDD and BD group. This vital evidence suggests that lymphocyte subsets may be effective biomarkers in distinguishing BD from MDD based on neuro-inflammation involved in the pathogenesis of BD and MDD. Based on the above, to assess the levels of MDD and BD lymphocyte subtypes in more detail, the potential factors for identifying MDD and BD were further explored in conjunction with clinical features and were shown in Supplementary Table 2. A total of 166 participants with BD and MDD were ultimately analysis, with more females than males in both groups. Participants with BD were younger in median onset age (25.74 years BD versus 33.39 years MDD), longer illness duration (136 months BD versus 36 months MDD), and higher median HAMD score (25 points BD versus 24 points MDD) compared to participants with MDD, and all three factors were statistically significant. In addition, there were nine factors of lymphocyte subsets that were significantly different between the MDD and BD groups, included CD19^+^ B cell counts, CD3^+^ T cell counts, CD3^+^/CD4^+^ T-helper cell counts, CD3^+^/CD4^+^ T-helper cell (%), CD3^+^/CD8^+^ T cell counts, CD3^+^/CD8^+^ T cell (%), CD3^–^CD16/56^+^ NK cell counts, CD4^+^CD8^+^ (ratio), and total lymphocyte counts. There are no factors with missing data.

### Feature selection

A total of twelve factors with statistically significant differences in Supplementary Table 2 were used as the original model and selected by LASSO regression. And based on the principle of the most regularized and stabilized model, the optimal parameter (lambda) through 10-fold cross-validation with minimum criteria was chosen. Then, the twelve factors were later reduced to eleven factors, including onset age, illness duration, HAMD score, CD19^+^ B cell counts, CD3^+^ T cell counts, CD3^+^/CD4^+^ T cell counts, CD3^+^/CD8^+^ T cell counts, CD3^+^/CD4^+^ T cell (%), CD3^–^CD16/56^+^ NK cell counts, CD4^+^CD8^+^ (ratio), and total lymphocyte counts, all with non-zero coefficients for the above. A cross-validated plot and a coefficient plot regarding the LASSO regression model are detailed in Supplementary Figures 1A,B.

### Development of differential diagnosis model

The univariate and multivariate logistic regression was used to analyze further the eleven factors assessed by the LASSO regression model, with the findings reported in Supplementary Table 3. The final analysis revealed four independent predictors that were strongly associated with the diagnosis of BD and MDD, including CD19^+^ B cell counts (OR, 1.109; 95% CI, 1.068–1.152), CD3^+^ T cell counts (OR, 1.107; 95% CI, 1.065–1.150), CD3^–^CD16/56^+^ NK cell counts (OR, 1.120; 95% CI, 1.074–1.168), total lymphocyte counts (OR, 0.904; 95% CI, 0.871–0.939), and shown in a forest plot (Supplementary Figure 2). The AIC for the multivariable model was 117.06. Based on this, a differential diagnosis model with those mentioned above independent four predictors (*P*-value < 0.05) were created and displayed as a ROC curve (Supplementary Figure 3) and nomogram (Supplementary Figure 4). The nomogram had an excellent discriminating ability with a high AUC of 0.922, the optimum cut-off value of this nomogram was 0.602, the sensitivity of 0.904, and specificity of 0.843. The nomogram shows the risk probability of each predictor from the multivariable model on the differential diagnosis of BD and MDD at the endpoint.

### Performance and clinical utility of differential diagnosis nomogram

The curve shape of the calibration plot indicated that the differential diagnosis nomogram was well-calibrated (Supplementary Figure 5A), with a non-significant Hosmer–Lemeshow test result confirming the goodness-of-fit of the model (χ^2^ = 9.471, *P*-value = 0.395). The AUC of the nomogram was 0.922 (95% CI, 0.879–0.965) and was confirmed as 0.911 (95% CI, 0.838–0.844) after internal validation, indicating that the model had an excellent discriminatory ability. The apparent performance of the differential diagnosis nomogram demonstrated a good prediction ability for BD and MDD. The DCA for the differential diagnosis nomogram was shown in Supplementary Figure 5B. The DCA result demonstrated that the threshold probability of more than 0.05, with a substantial benefit for distinguishing between the BD and MDD groups.

## Discussion

### An overview of the findings

In the current study, given that blood sample collection is an easy-to-acquire technique, we used a flow cytometric analysis technique to quantify factors of lymphocyte subtypes from participants with BD and MDD as well as compare them with HCs. Finally, it was concluded that lymphocyte subtypes play an important role in neural pathways during depressive episodes in participants with MDD and BD as an indicator of status. Based on this, we applied the machine learning technique to identify four independent predictors in lymphocyte subtypes strongly associated with BD and MDD diagnosis. Simultaneously, a differential diagnosis nomogram had a discriminatory ability (C-index, 0.922) was developed and validated. According to our knowledge, this is the first study to provide valuable evidence that CD19^+^ B cell counts, CD3^+^ T cell counts, CD3^–^CD16/56^+^ NK cell counts, and total lymphocyte counts were diagnostic markers for individualized prediction of BD and MDD, which paves the way for the subsequent selection of appropriate clinical treatment strategies (e.g., drug selection) to guarantee a favorable prognosis and avoid unwanted outcomes.

### Meaningful clinical features linked with the diagnosis of BD and MDD

In the univariate model of our study, onset age, illness duration, and HAMD score were associated with the diagnosis of BD and MDD, consistent with previous studies (50–52). Even though these factors were not included in the final nomogram, their clinical significance cannot be disregarded. It can be noted that the median onset age of the BD group was 7.65 years earlier than the MDD group in our cohort. This was closed to the findings of Tondo et al., who studied the onset age of BD and MDD from a cohort combined with 3,014 mood disorder patients, and found that the median onset age for the two disorders was 23 and 32 years, respectively, a difference of about 9 years. (53). It is widely believed that onset age helps differentiate BD from MDD, the typical onset of BD takes place from late adolescence to early adulthood (54). At the same time, MDD is more inclined to later ages onset (55). On the whole, BD patients are of earlier onset age, longer illness duration (56), with more severe clinical presentation and poorer outcomes (such as more episodes of depression, greater severity of depression) (51, 56).

### Intriguing lymphocyte subsets are strongly associated with the diagnosis of BD and MDD

Based on multivariate logistic regression, the nomogram highlighted the four predictors used to differentiate BD from MDD based on lymphocyte subtypes: higher CD19^+^ B cell counts, CD3^+^ T cell counts, CD3^–^CD16/56^+^ NK cell counts, and lower total lymphocyte counts. Our nomogram illustrates how these various predictors can be combined to help predict the likelihood of BD. For example, for a participant with CD19^+^ B cell counts, CD3^+^ T cell counts, CD3^–^CD16/56^+^ NK cell counts, and total lymphocyte counts of 424.8, 2,056.6, 233.4, and 2,706 (cells/1 μl), respectively, the risk of being diagnosed with BD would be approximately 92.9%.

For elevated CD19^+^ and CD3^+^ cell counts in BD patients than MDD patients of our nomogram, and the results of the pairwise comparison further showed that CD19^+^ B cells were higher in both BD and MDD than in HCs; this result is consistent with previous studies exploring the comparison of CD19^+^ levels in BD or MDD with HCs (30, 31). Interestingly, our study compared the difference in CD19^+^ levels between BD and MDD, with MDD being lower than BD. Moreover, patients with MDD had decreased CD3^+^ T cells, whereas patients with BD had greater CD3^+^ cell levels than HCs, and BD than MDD. As stated by Karlijn et al., the absence of T cells in MDD and a normal or overactive T cell system may be characteristic of BD (19). Therefore, the CD3^+^ T cells and CD19^+^ B cells become diagnosis indicators of BD and MDD, which relevant T lymphocyte paradigm may help explain the finding. There is evidence that T cells activation has indeed been highlighted as a possible trait in BD patients (57), and is a response to the chronic low inflammatory state caused by an excess of immune modulation which inducing relevant symptoms (58), particularly the elevation level of activated CD3^+^ T cells (22). Later findings have shown that an increased proportion of activated T cells and a trend in T helper 2 (Th2) activation in BD patients (22, 26). Moreover, there is a cognate interaction between B cells and T cells (59), B cells do not exhibit regulatory functions (60) due to the lack of major histocompatibility complex class II and B7 (61, 62), however, the activation of Th2 from T cell creates the conditions for activating B cells and the production of autoantibodies, further increased CD19^+^ levels (22). In this line, it shows that the Th2 cell play a crucial role as a hub. Leday et al. suggested that MDD is associated with the downregulation of genes related to T lymphocyte function and adaptive immunity (63). On the one hand, it may be due to the lack of Th2 expressed by T cells, therefore, the inability to activate B cells, resulting in mildly reduced or near-normal CD19^+^ levels. On the other hand, it is the lack of T lymphocytes and the inability to induce f interleukin (IL)-10 in the meninges and prefrontal cortex (PFC); hence, a genetic predisposition to produce less IL-10 is associated with a higher risk for depressive symptoms (64).

Compared to MDD, NK levels were higher in BD, but both were below normal levels compared to HCs, all of which are supported by the results of previous studies. Studies revealed that patients with BD exhibit a decrease in NK cells when they experience mania as compared to HCs. (65), whereas in outpatients with stable BD, there was no change in the number of NK cells compared to HCs (22). Furthermore, MDD may be associated with reduced natural killer cell activity (NKCA) and NK cell counts, especially early-onset-age MDD (66). The reason for this discrepancy between BD and MDD suggests that an interaction between affective disorders and various parameters of the immune system may be associated with altered immunity (65, 66). It is proposed that chronic pro-inflammatory processes in individuals with MDD may directly affect NK cells, suppressing NK cell function and numbers (67). We all know that when depression occurs in patients with BD, it is accompanied by the onset of mania. Tsai et al. demonstrated the presence of cell-mediated activation when BD patients experience mania. They found that, compared to HCs, BD patients with the manic had a proliferation of lymphocytes responding to phytohemagglutinin (PHA), and soluble interleukin-2R receptor (sIL-2R) was released from activated T cells into the blood, leading to a significant increase in plasma sIL-2R levels, thereby reducing cellular immune function (68).

Interestingly, some studies thought lymphopenia is a common observation in patients with BD and MDD (25, 64). Nevertheless, not absolute, Denis et al. presented a study on the alterations in neutrophils and lymphocytes in the blood of mood disorder patients, and discovered that lymphopenia occurs solely in MDD and not in BD (69), which is the same as our conclusion. We found significantly fewer total lymphocyte counts in MDD compared with HCs, but no difference was found in BD; and total lymphocyte counts in BD were more significant than in MDD, which is consisted with the study of Abeer et al. (65). In addition, combining the results of multivariate logistic regression further revealed that total lymphocyte count was negatively associated with the occurrence of BD and, conversely, positively associated with the occurrence of MDD. As shown by the results of our nomogram, individuals with lower total lymphocyte counts were more likely to be diagnosed with BD than MDD. As of now, the mechanisms of total lymphocyte count in mood disorders remain incompletely understood, and there is contradictory information concerning lymphocyte subsets and total lymphocyte counts in patients with BD and MDD (70). Insufficient numbers of circulating T helper cells and their subtypes in MDD and BD by previous scholars can lead to increased T cell apoptosis (19), which may further cause a decrease in total lymphocyte counts. Neuroinflammation mechanisms in patients with BD and MDD have been extensively described in terms of the levels of cytokines, HPA axis, neurotransmitters, and neurotrophic factors (71). Therefore, apoptosis may be associated with neuroinflammatory mechanisms, the findings of previous studies may provide some evidence to explain the difference between MDD and BD, which can be include as the following aspects.

(i) Patients with BD produce higher levels of pro-inflammatory cytokines during acute exacerbations due to the inhibition of T helper cell proliferation and function by tumor necrosis factor alpha (TNF-α) (72, 73). However, there is also a trend for elevated pro-inflammatory cytokine levels in MDD patients (74), which has been suggested that elevated pro-inflammatory cytokines could be responsible for the difference in T helper cell apoptosis in MDD and BD patients.

(ii) Differences in relevant pharmacological treatment strategies between MDD and BD. In particular, lithium or valproic acid do affect on cell apoptosis/proliferation (75, 76), and therefore it can be assumed that this class of drugs produces the effect. However, Ezequiel et al. found that when patients received any therapeutic dose of an emotional drug (lithium or valproic acid) or not, the proportion of lymphocytes exhibiting apoptosis was significantly increased in BD patients compared to HCs, which correlated with the high expression of Bax protein in patient cells (77). Similarly, Bei et al. found similar results in BD patients where increased lymphocyte apoptosis was caused by increased cytochrome C content in the cytoplasm and translocation of Bax protein (78). It can be seen that the difference between BD and MDD is not only limited to drug differences, but is more caused by the own endogenous substances of patients.

(iii) It may associate with tryptophan depletion. Tryptophan is a proliferation stimulator for T cells, and depletion of tryptophan has been shown to lead to decreased T cell proliferation and apoptosis of T cells (79). Activation of indoleamine-2,3-dioxygenase (IDO) causes the enhanced synthesis of tryptophan in MDD, leading to excessive degradation of tryptophan (80). However, low tryptophan levels are also present in BD patients (81). Therefore, tryptophan depletion needs to be explored in depth.

(iv) The abnormal state of microglia (microglia activation) and other immunocompetent cells in the brain is the driving force behind the phenomenon (82). Stertz et al. postulated that BD patients undergoing an acute emotional episode induces neuronal damage, which results in the discharge of damage-associated molecules that actuate microglia (83). Activated microglia can then generate pro-inflammatory cytokines and neurotrophic factors. These molecules cause alteration in the synaptic environment *via* synaptic pruning as an adaption attempt to the damage caused by acute episode. Then, after multiple repeated episodes, the overproduction of pro-inflammatory cytokines outstrips the normal down-regulatory ability to down-regulate them, causing microglia to remain constantly activated and inducing apoptosis failure to develop adequate (adaptive) stress-related responses (84, 85). Again, this phenomenon has been found to be present in patients with MDD. Studies have confirmed the ability of Th1 and Th17 cells to promote neuroinflammation and the activation of microglia and astrocytes (86, 87). In addition, high levels of T helper 1 (Th1) and T helper 17 (Th17) are often considered to be markers of inflammation, while high levels of T regulatory, and Th2 cells play an anti-inflammatory role. And Th17, T regulatory, and Th2 cells are all trending up in BD patients and conversely, down in MDD patients. In other words, it seems that both pro-inflammatory and anti-inflammatory forces are activated in BD, but both are suppressed in MDD, both of which contribute to a greater degree of apoptosis in BD than in MDD (19, 88–90).

### Satisfactory performance for differential diagnosis nomogram

This study is based on a novel and widely used methodologies in mental disorders-machine learning techniques, including the LASSO analysis method and nomogram has the ability to superimpose the relative risk of important features and improve its predictive power (91), and eliminate interactions between features (92). Nomogram as a distinguishable predictive tool, not only correctly determines disease conditions based on the level of connection between features and diseases, but also delivers targeted risk assessments for the corresponding disease groups by building risk thresholds for diagnostic decisions (93, 94). Based on the selected features, we constructed a differential diagnosis nomogram that can serve as a feasible scoring system. We obtained a good verification result through the bootstrapping method (C-index, 0.911) and the calibration curve and DCA curve also exhibited favorable agreement and applicability, respectively. In view of our findings, all these predictors could be low-economical and easily obtained in the blood collection, which is of great significance for patients with mood disorders. Additionally, clinical staff can use this individualized diagnostic model to diagnose the probability of BD and MDD, which aligns with the current concept of precision medicine (95).

### Strength

One of the strengths of this study is that as a prospective study, it facilitated us to design stringent diagnostic, inclusion, and exclusion criteria for an accurate distinction between BD and MDD to guarantee the authenticity and reliability of the data source. Another strength is our study based on machine learning techniques to explore CD19^+^ B cell counts, CD3^+^ T cell counts, CD3^–^CD16/56^+^ NK cell counts, and total lymphocyte counts as new predictors to differentiate BD and MDD.

### Limitation and future directions

Although we present a helpful tool for distinguishing between BD and MDD, there are not, however, devoid of limitations. Firstly, the recruitment of participants for our study was performed with a small sample of participants who had BD and MDD at a single center. Although the internal validation of nomograms has good performance, it is still debatable whether nomograms have extrapolation power, and further expansion of sample size or replication in combination with multicenter is needed. Secondly, on the one hand, our nomogram was developed with the clinical features and the characteristic of lymphocyte subsets. The clinical features should be further refined, such as obesogenic diet and medication use; in addition to lymphocyte subsets, immunophenotyping and plasma cytokine should be added. On the other hand, incorporating the computed tomography (CT), electroencephalographic (EEG), and magnetic resonance imaging (MRI) information; combining the above additional variables to continue the prospective data collection effort could optimize our current nomogram. Thirdly, we did not collect blood samples after follow-up, so in future studies, we will build a follow-up database to obtain more dynamic changes in lymphocyte subtype levels and further investigate the dynamic role of lymphocyte subtypes in the neuroinflammatory pathways of BD and MDD. Fourth, from Müller et al., there is heterogeneity in patients with BD and MDD, leading to conflicting neuro-immunological findings in patients with affective disorders, especially related to different depression subtypes (29). The participants recruited in this study included both first-episode and relapsed BD and MDD participants and did not distinguish between BD and MDD subtypes to explore differences in lymphocyte subtypes, which could lead to selection bias and may affect the study results, which requires refinement of the study protocol in future studies by designing strict inclusion criteria further explore the differences between first-episode BD and MDD patients, relapsed BD and MDD patients, BD subtypes and MDD subtypes patients between the lymphocyte subtype levels, and to verify whether nomograms have similar diagnostic power when explored in the above stratification. Finally, all participants are likely to be treated with mood stabilizing medications or antidepressants, which inevitably impact the regulation of immune function. Therefore, the next step should be to collect detailed information about medication use of participants and explore changes in lymphocyte subtypes under drug stratification.

## Conclusion

Our findings suggest that lymphocyte subsets (CD19^+^ B cell count, CD3^+^ T cell count, CD3^–^CD16/56^+^ NK cell count, and total lymphocyte count) play an important role in the pathogenesis of BD and MDD as an indicator of biological status, especially during depressive episodes. As blood samples are a simple and economical test that is routinely performed in patients with BD and MDD, investment in this line of research may lead to the discovery of a firmer link between biological parameters and psychopathological indicators of BD and MDD. These complex biomarkers of affective disorders could in turn facilitate the identification of new therapeutic strategies.

Furthermore, the differential diagnosis nomogram developed in our study based on machine learning techniques is well calibrated and discriminatory and could be an easy-to-use, repeatable, and economical diagnostic tool to help clinicians differentiate between BD and MDD. This also demonstrates that machine learning approaches are very effective in dealing with the interactions between biomarkers of neuroinflammatory disorders. Therefore, based on the existing findings and previous research findings, we are confident enough to expect that machine learning party techniques will play a more effective role in disease neuroinflammation research and model building in BD and MDD compared to traditional statistical analysis methods, which may be a pleasing trend in the future.

## Data availability statement

The original contributions presented in this study are included in the article/Supplementary material, further inquiries can be directed to the corresponding authors.

## Ethics statement

The studies involving human participants were reviewed and approved by the Ethics Committee of Huzhou Third Municipal Hospital. The patients/participants provided their written informed consent to participate in this study.

## Author contributions

ZS, LS, and XS: study concept and design. XZ, YBS, YS, and SM: data collection. LS and YS: statistical analysis of the data. LS and ZS: manuscript preparation. ZS, XS, and YBS: critical revision of the manuscript. All authors contributed to the article and approved the submitted version.

## Footnotes

1
https://cran.r-project.org/web/packages
