# Supplementary material for: Development and validation of a nomogram based on lymphocyte subsets to distinguish bipolar depression from major depressive disorder
Source: Front Psychiatry. 2022 Oct 6;13:1017888. doi: 10.3389/fpsyt.2022.1017888 (PMC9583168; doi:10.3389/fpsyt.2022.1017888)
Supplement: Supplementary file 1 [file Data_Sheet_1.docx]

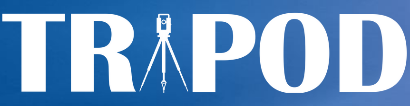
***SUPPLEMENTARY MATERIAL 1***

# SUPPLEMENTARY MATERIAL 1 - The TRIPOD Checklist: Prediction Model Development.

| **Section/Topic** | **Item** | **Checklist Item** | **Reported on**  **Page Number/Line Number** | **Reported on Section/Paragraph** |
| --- | --- | --- | --- | --- |
| **Title and abstract** | | | | |
| Title | 1 | Identify the study as developing and/or validating a multivariable prediction model, the target population, and the outcome to be predicted. | Page 1/Shown in the title | Title page/Shown in the title |
| Abstract | 2 | Provide a summary of objectives, study design, setting, participants, sample size, predictors, outcome, statistical analysis, results, and conclusions. | Page 1/Line 11-35 | Abstract/Para 1-4 |
| **Introduction** | | | | |
| Background  and objectives | 3a | Explain the medical context (including whether diagnostic or prognostic) and rationale for developing or validating the multivariable prediction model, including references to existing models. | Page 2-3/Line 36-98 | Introduction/Para 1-3 |
|  | 3b | Specify the objectives, including whether the study describes the development or validation of the model or both. | Page 3/Line 99-104 | Introduction/Para 4 |
| **Methods** | | | | |
| Source of data | 4a | Describe the study design or source of data (e.g., randomized trial, cohort, or registry data), separately for the development and validation data sets, if applicable. | Page 3/Line 107-108 | Materials and methods/Para 1 |
|  | 4b | Specify the key study dates, including start of accrual; end of accrual; and, if applicable, end of follow-up. | Page 3/Line 107-108 | Materials and methods/Para 1 |
| Participants | 5a | Specify key elements of the study setting (e.g., primary care, secondary care, general population) including number and location of centres. | Page 3/Line 107-108 | Materials and methods/Para 1 |
|  | 5b | Describe eligibility criteria for participants. | Page 3/Line 112-120 | Materials and methods/Para 2 |
|  | 5c | Give details of treatments received, if relevant. | Not applicable | This is only a diagnostic |
| Outcome | 6a | Clearly define the outcome that is predicted by the prediction model, including how and when assessed. | Page 4/Line 126-154 | Materials and methods/Para 4-7 |
|  | 6b | Report any actions to blind assessment of the outcome to be predicted. | Page 4/Line 141-142 | Materials and methods/Para 6 |
| Predictors | 7a | Clearly define all predictors used in developing or validating the multivariable prediction model, including how and when they were measured. | Page 4/Line 125-153 | Materials and methods/Para 4-7 |
|  | 7b | Report any actions to blind assessment of predictors for the outcome and other predictors. | Page 4/Line 142-143 | Materials and methods/Para 6 |
| Sample size | 8 | Explain how the study size was arrived at. | Page 3/Line 109-111 | Materials and methods/Para 1 and 3 |
| Missing data | 9 | Describe how missing data were handled (e.g., complete-case analysis, single imputation, multiple imputation) with details of any imputation method. | Page 5/Line 191-192 | Results/Para 1 |
| Statistical analysis methods | 10a | Describe how predictors were handled in the analyses. | Page 4-5/Line 155-176 | Materials and methods/Para 8-9 |
|  | 10b | Specify type of model, all model-building procedures (including any predictor selection), and method for internal validation. | Page 5/Line 177-188 | Materials and methods/Para 10-11 |
|  | 10d | Specify all measures used to assess model performance and, if relevant, to compare multiple models. | Page 5/Line 177-188 | Materials and methods/Para 10-11 |
| Risk groups | 11 | Provide details on how risk groups were created, if done. | Not applicable | No risk groups |
| **Results** | | | | |
| Participants | 13a | Describe the flow of participants through the study, including the number of participants with and without the outcome and, if applicable, a summary of the follow-up time. A diagram may be helpful. | Page 5/Line 193 | Results/Para 1 |
|  | 13b | Describe the characteristics of the participants (basic demographics, clinical features, available predictors), including the number of participants with missing data for predictors and outcome. | Page 5/Line 190-201 | Results/Para 1 |
| Model development | 14a | Specify the number of participants and outcome events in each analysis. | Page 5/Line 193 | Results/Para 1 |
|  | 14b | If done, report the unadjusted association between each candidate predictor and outcome. | Not applicable | Not done |
| Model specification | 15a | Present the full prediction model to allow predictions for individuals (i.e., all regression coefficients, and model intercept or baseline survival at a given time point). | Page 6/Line 212-219 | Results/Para 3 |
|  | 15b | Explain how to the use the prediction model. | Page 6/Line 220-237 | Results/Para 3-4 |
| Model performance | 16 | Report performance measures (with CIs) for the prediction model. | Page 6/Line 223-234 | Results/Para 3-4 |
| **Discussion** | | | | |
| Limitations | 18 | Discuss any limitations of the study (such as nonrepresentative sample, few events per predictor, missing data). | Page 9/Line 354-374 | Discussion/Para 13 |
| Interpretation | 19b | Give an overall interpretation of the results, considering objectives, limitations, and results from similar studies, and other relevant evidence. | Page 6-9/Line 238-374 | Discussion/Para 1-13 |
| Implications | 20 | Discuss the potential clinical use of the model and implications for future research. | Page 6/Line 245-248, and Page 9/Line 343-353;  Page 9/Line 375- 380 | Discussion/Para 1, 12; Conclusion/Para 1 |
| **Other information** | | | | |
| Supplementary information | 21 | Provide information about the availability of supplementary resources, such as study protocol, Web calculator, and data sets. | Page 10/Line 383-385 | Data availability statement/Para 1 |
| Funding | 22 | Give the source of funding and the role of the funders for the present study. | Page 10/Line 395-398 | Funding/Para 1 |
