# Supplementary material for: Development and validation of a nomogram based on lymphocyte subsets to distinguish bipolar depression from major depressive disorder
Source: Front Psychiatry. 2022 Oct 6;13:1017888. doi: 10.3389/fpsyt.2022.1017888 (PMC9583168; doi:10.3389/fpsyt.2022.1017888)
Supplement: Supplementary file 2 [file Data_Sheet_2.docx]

***SUPPLEMENTARY MATERIAL 2***

# SUPPLEMENTARY MATERIAL 2 - Table 1

. **Table 1 Gender, age, and lymphocyte subsets characteristics of the groups**

| Factors | N (%) for binary measures,  and Mean (S.D.) / Median (IQR) for quantitative variables | | | F/H/${}^{2}$ | P -value | Pairwise comparisons |
| --- | --- | --- | --- | --- | --- | --- |
|  | HCs Group  (n = 101) | MDD Group  (n = 83) | BD Group  (n = 83) |  |  |  |
| Gender | | | | | | |
| Male | 30 (29.70) | 17 (20.48) | 23 (27.71) | 2.13 | None | None |
| Female | 71 (69.30) | 66 (79.52) | 60 (72.29) |  |  |  |
| Age (years) ^c #^ | 38.00 (16.00) | 41.00 (15.00) | 37.00 (22.00) | 0.34 | None | None |
| CD19^+^ (cells/μl) ^c #^ | 213.50 (93.35) | 261.20 (167.20) | 310.80 (178.60) | 46.19 | < 0.0001 | BD > MDD > HCs |
| CD19^+^ (%)^c #^ | 11.00 (4.80) | 13.90 (6.50) | 15.30 (5.40) | 50.08 | < 0.0001 | BD > HCs; MDD > HCs |
| CD3^+^ (cells/μl) ^c #^ | 1437.4 (629.70) | 1334.10 (459.90) | 1640.6 (522.20) | 23.02 | < 0.0001 | BD > HCs > MDD |
| CD3^+^ (%) ^c #^ | 68.90 (11.10) | 75.50 (8.30) | 75.50 (7.90) | 43.13 | < 0.0001 | BD > HCs > MDD |
| CD3^+^/CD4^+^ (cells/μl) ^c #^ | 711.70 (373.75) | 866.50 (326.00) | 928.60 (365.80) | 26.87 | < 0.0001 | BD > MDD > HCs |
| CD3^+^/CD4^+^ (%) ^b $^ | 33.73 (7.11) | 47.05 (8.19) | 43.12 (5.58) | 88.15 | < 0.0001 | MDD > BD> HCs |
| CD3^+^/CD8^+^ (cells/μl) ^c #^ | 528.70 (256.20) | 393.40 (257.60) | 589.40 (266.40) | 35.49 | < 0.0001 | HCs > BD; BD > MDD |
| CD3^+^/CD8^+^ (%) ^c #^ | 25.50 (7.65) | 23.50 (8.50) | 28.00 (7.10) | 15.11 | 0.001 | BD > MDD |
| CD3^-^CD16/56^+^(cells/μl) ^c #^ | 437.30 (288.60) | 155.40 (118.40) | 205.30 (141.30) | 105.58 | < 0.0001 | HCs > BD > MDD |
| CD3^-^CD16/56^+^ (%) ^c #^ | 20.30 (10.25) | 9.20 (7.20) | 8.50 (5.70) | 108.01 | < 0.0001 | HCs > MDD; HCs > BD |
| CD4^+^CD8^+^ (ratio) ^c #^ | 1.16 (0.57) | 2.08 (1.10) | 1.54 (0.56) | 73.09 | < 0.0001 | MDD > BD > HCs |
| DN T (%) ^c #^ | 7.60 (5.50) | 4.40 (4.90) | 5.70 (3.80) | 19.62 | < 0.0001 | HCs > MDD; HCs > BD |
| Total lymphocyte count (cells/μl) ^c #^ | 2314.00 (541.00) | 1763.00 (626.00) | 2193.00 (717.00) | 46.17 | < 0.0001 | HCs > MDD; BD > MDD |
| **Notes:** ^a^ Chi-square tests [Number (percentage), N (%)]; ^b^ Univariate ANOVA [Mean (Standard Deviation), Mean (S.D.)]; ^c^ Kruskal-Wallis H test [(Median (Interquartile range), Median (IQR)).  P-value: Omnibus p-values with Kruskal-Wallis H test or univariate ANOVA for multiple comparisons; ^#^ Nemenyi correction of Kruskal-Wallis H test of pairwise comparisons, ^$^ Bonferroni correction of univariate ANOVA of pairwise comparisons.  P-values < 0.05 was considered statistically significant. | | | | | | |

# SUPPLEMENTARY MATERIAL 2 - Table 2

**Table 2** **Lymphocyte subsets and participant's characteristics comparing MDD** **versus BD**

| Factors | Overall  (n = 166) | MDD Group  (n = 83) | BD Group  (n = 83) | P-value |
| --- | --- | --- | --- | --- |
| **Clinical features** | | | | |
| Gender ^a^ | | | | |
| Male | 40 (24.10) | 17 (20.48) | 23 (27.71) | 0.364 |
| Female | 126 (75.90) | 66 (79.52) | 60 (72.29) |  |
| Age (years) ^c^ | 39.10 (11.86) | 41.00 (15.00) | 37.00 (22.00) | 0.770 |
| Weight (kg) ^c^ | 60.24 (12.94) | 56.00 (13.60) | 58.60 (17.80) | 0.183 |
| Height (m) ^c^ | 1.62 (0.07) | 1.60 (0.08) | 1.62 (0.10) | 0.160 |
| BMI (kg/m^2^) ^b^ | 22.78 (3.97) | 22.51 (3.52) | 23.05 (4.37) | 0.369 |
| Onset age (years) ^b^ | 29.56 (10.89) | 33.39 (10.48) | 25.74 (9.96) | **< 0.0001** |
| Illness duration (month) ^c^ | 114.51 (114.38) | 36.00 (99.00) | 136.00 (140.00) | **< 0.0001** |
| Current episode duration (month) ^c^ | 10.12 (25.74) | 2.00 (5.00) | 3.00 (10.00) | 0.161 |
| Smoking ^a^ | | | | |
| Yes | 36 (21.70) | 19 (22.89) | 17 (20.48) | 0.850 |
| No | 130 (78.30) | 64 (77.11) | 66 (79.52) |  |
| Drinking ^a^ | | | | |
| Yes | 44 (26.50) | 21 (25.30) | 23 (27.71) | 0.861 |
| No | 122 (73.50) | 62 (74.71) | 60 (72.29) |  |
| Education level ^a^ | | | | |
| ≤5 years | 29 (17.50) | 8 (9.64) | 21 (25.30) | 0.062 |
| 6-8 years | 63 (38.00) | 36 (43.37) | 27 (32.53) |  |
| 9-12 years | 40 (24.1) | 21 (25.30) | 19 (22.89) |  |
| ≥12 years | 34 (20.5) | 18 (21.69) | 16 (19.28) |  |
| Employment status ^a^ | | | | |
| Employed | 70 (42.20) | 39 (46.99) | 31 (37.35) | 0.271 |
| Unemployed | 96 (57.80) | 44 (53.01) | 52 (62.65) |  |
| Marital status ^a^ | | | | |
| Married | 69 (41.60) | 37 (44.58) | 32 (38.55) | 0.529 |
| Single/Divorce/Widowed | 97 (58.40) | 46 (55.42) | 51 (61.45) |  |
| Family history of affective disorders ^a^ | | | | |
| Yes | 21(12.70) | 9 (10.84) | 12 (14.46) | 0.641 |
| No | 145 (87.3) | 74 (89.16) | 71 (85.54) |  |
| HAMA score ^c^ | 23.58 (2.68) | 24.00 (2.00) | 24.00 (1.00) | 0.115 |
| HAMD score ^c^ | 24.89 (2.78) | 24.00 (3.00) | 25.00 (3.00) | **0.007** |
| **The characteristics of lymphocyte subsets** | |  |  |  |
| CD19^+^ (cells/μl) ^c^ | 319.45 (158.15) | 261.20 (167.20) | 310.80 (178.60) | **< 0.0001** |
| CD19^+^ (%) ^c^ | 15.21 (4.48) | 13.90 (6.50) | 15.30 (5.40) | 0.144 |
| CD3^+^ (cells/μl) ^c^ | 1521.05 (445.96) | 1334.10 (459.90) | 1640.6 (522.20) | **< 0.0001** |
| CD3^+^ (%) ^c^ | 74.16 (6.98) | 75.50 (8.30) | 75.50 (7.90) | 0.505 |
| CD3^+^/CD4^+^ (cells/μl) ^c^ | 920.48 (288.52) | 866.50 (326.00) | 928.60 (365.80) | **0.008** |
| CD3^+^/CD4^+^ (%) ^b^ | 45.09 (7.26) | 47.05 (8.19) | 43.12 (5.58) | **< 0.0001** |
| CD3^+^/CD8^+^ (cells/μl) ^c^ | 530.65 (212.54) | 393.40 (257.60) | 589.40 (266.40) | **< 0.0001** |
| CD3^+^/CD8^+^ (%) ^c^ | 25.75 (6.87) | 23.50 (8.50) | 28.00 (7.10) | **< 0.0001** |
| CD3^-^CD16/56^+^(cells/μl) ^c^ | 212.90 (135.98) | 155.40 (118.40) | 205.30 (141.30) | **< 0.0001** |
| CD3^-^CD16/56^+^ (%) ^c^ | 9.87 (6.01) | 9.20 (7.20) | 8.50 (5.70) | 0.796 |
| CD4^+^CD8^+^ (ratio) ^c^ | 1.93 (0.79) | 2.08 (1.10) | 1.54 (0.56) | **< 0.0001** |
| DN T (%) ^c^ | 36.68 (166.83) | 4.40 (4.90) | 5.70 (3.80) | 0.134 |
| Total lymphocyte count (cells/μl) ^c^ | 2055.42 (590.44) | 1763.00 (626.00) | 2193.00 (717.00) | **< 0.0001** |
| **Notes:** ^a^ Fisher's exact test/ Chi-square test [Number (percentage), N (%)]; ^b^ Independent t-test [Mean (Standard Deviation), Mean (S.D.)]; ^c^ Mann-Whitney U test [(Median (Interquartile range), Median (IQR)); m, meter; BMI, body mass index. P-values < 0.05 was considered statistically significant. | | | | |

# SUPPLEMENTARY MATERIAL 2 - Table 3

**Table 3** **Univariable and multivariable logistic regression analysis of BD diagnosis**

| Predictors | Univariable analysis | |  | | Multivariable analysis | |
| --- | --- | --- | --- | --- | --- | --- |
|  | OR (95% CI) | P-value | | OR (95% CI) | | P-value |
| Onset age (years) | 0.929 (0.899-0.961) | **< 0.0001** | | 0.973 (0.921-1.028) | | 0.325 |
| Illness duration (month) | 1.010 (1.005-1.014) | **< 0.0001** | | 1.007 (1.000-1.013) | | 0.051 |
| HAMD score | 1.182 (1.048-1.333) | **0.006** | | 1.177 (0.943-1.469) | | 0.149 |
| CD19^+^(cells/μl) | 1.004 (1.002-1.007) | **0.0001** | | 1.109 (1.068-1.152) | | **< 0.0001** |
| CD3^+^ (cells/μl) | 1.002 (1.001-1.003) | **< 0.0001** | | 1.107 (1.065-1.150) | | **< 0.0001** |
| CD3^+^/CD4^+^ (%) | 0.922 (0.880-0.967) | **0.0001** | | 1.092 (0.902-1.323) | | 0.366 |
| CD3^-^CD16/56^+^ (cells/μl) | 1.004 (1.001-1.007) | **0.009** | | 1.120 (1.074-1.168) | | **< 0.0001** |
| CD4^+^CD8^+^ (ratio) | 0.399 (0.245-0.651) | **< 0.0001** | | 0.158 (0.022-1.146) | | 0.068 |
| Total lymphocyte count (cells/μl) | 1.001 (1.001-1.002) | **< 0.0001** | | 0.904 (0.871-0.939) | | **< 0.0001** |
| **Notes:** OR, odds ratio; CI, confidence interval. P-values < 0.05 was considered statistically significant. | | | | | | |

# SUPPLEMENTARY MATERIAL 2 - Table 4

**Table 4 Abbreviations**

| **Abbreviations** | |
| --- | --- |
| BD | Bipolar Depression |
| MDD | Major Depressive Disorder |
| HCs | Healthy Controls |
| LASSO | Least Absolute Shrinkage and Selection Operator |
| ROC | Receiver Operating Characteristic |
| AUC | Area Under the Curve |
| C-index | Harrell’s Concordance index |
| DCA | Decision Curve Analysis |
| SPSS | Statistical Package for the Social Sciences |
| glmnet | Lasso and Elastic-Net Regularized Generalized Linear Models |
| pROC | Display and Analyze ROC Curve |
| rms | Regression Modeling Strategy |
| rmda | Perform DCA plot |
| generalhoslem | Perform the Hosmer-Lemeshow test for detected goodness-of-fit |
| CI | Confidence Interval |
| OR | Odds Ratio |
| AIC | Akaike Information Criterion |
| SCID | Structural Clinical Interview for DSM-IV Disorder |
| DSM-IV | Diagnostic and Statistical Manual of Mental Disorders-IV |
| HAM-D_17_ | 17-item Hamilton Rating Scale for Depression |
| HAMA | Hamilton Rating Scale for Anxiety |
| BMI | Body Mass Index |
| TRIPOD | Transparent Reporting of a Multivariable Prediction Model for Individual Prognosis or Diagnosis |
| B | Breg |
| T | Treg |
| NK | Natural Killer |
| DN T | Double Negative T cell |
| CD19^+^ | CD19^+^ B cell count |
| CD19^+^ (%) | CD19^+^ T cell percentage |
| CD3^+^ | CD3^+^ T cell count |
| CD3^+^ (%) | CD3^+^ T cell |
| CD3^+^/CD4^+^ | CD3^+^/CD4^+^ T-helper cell count |
| CD3^+^/CD4^+^ (%) | CD3^+^/CD4^+^ T-helper percentage |
| CD3^+^/CD8^+^ | CD3^+^/CD8^+^ T-cytotoxic cell count |
| CD3^+^/CD8^+^ (%) | CD3^+^/CD8^+^ T-cytotoxic cell percentage |
| CD3^-^CD16/56^+^ | CD3^-^CD16/56^+^ NK cell count |
| CD3^-^CD16/56^+^ (%) | CD3^-^CD16/56^+^ Percentage of NK cell |
| CD4^+^CD8^+^ (ratio) | CD3^+^/CD4^+^ T-helper cell count to CD3^+^/CD8^+^ T-cytotoxic cell count ratio |
| CD3^+^CD4^-^CD8^-^ T cell (%) | TCRαβ^+^ CD3^+^CD4^-^CD8^-^ double negative T cell percentage |
