# Supplementary material for: Development and validation of a nomogram based on lymphocyte subsets to distinguish bipolar depression from major depressive disorder
Source: Front Psychiatry. 2022 Oct 6;13:1017888. doi: 10.3389/fpsyt.2022.1017888 (PMC9583168; doi:10.3389/fpsyt.2022.1017888)
Supplement: Supplementary file 3 [file Data_Sheet_3.docx]

***SUPPLEMENTARY MATERIAL 3***

# SUPPLEMENTARY MATERIAL 3 - Figure. 1

**
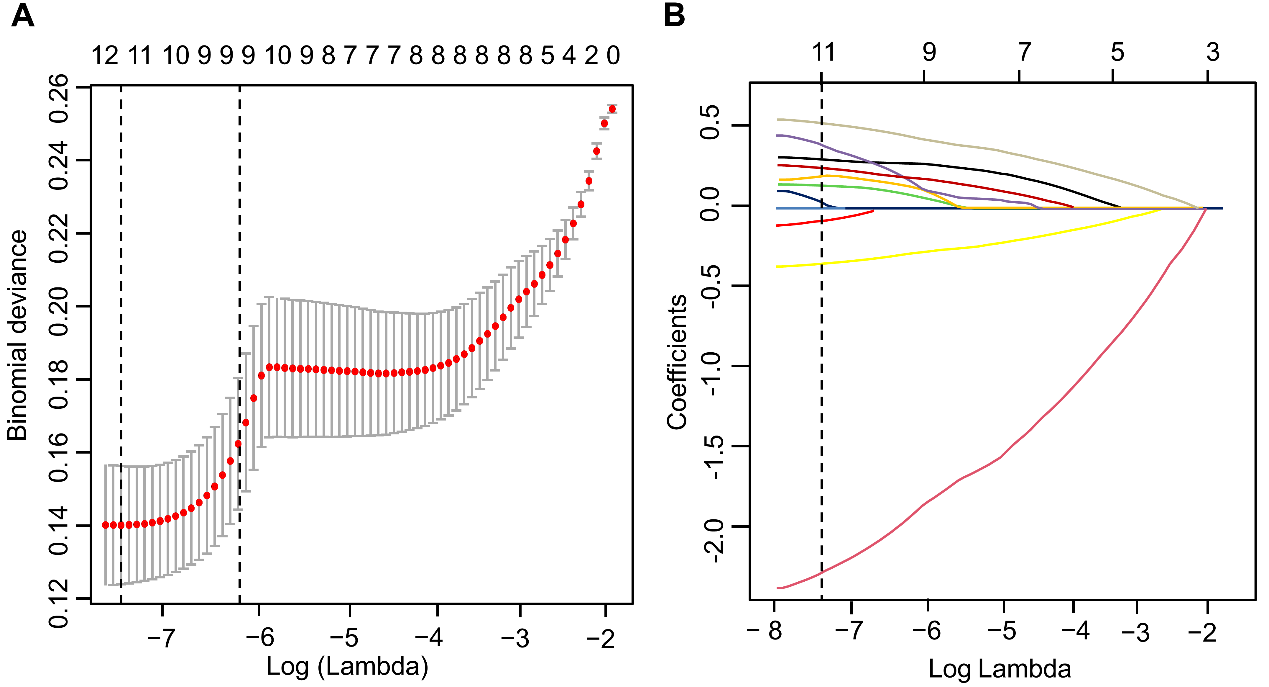
**

**Figure. 1** The LASSO regression model was used to select significant factors. **(A)**The optimal parameter (Lambda) identified in the LASSO model was used with 10-fold cross-validation and minimum criteria. The left dotted vertical line represented the minimum criteria, and the right vertical line represented the 1 SE of the minimum criteria (the 1-SE criteria). Consequently, a Lambda value of 0.00058 was chosen, with Log (Lambda) = -7.45. **(B)** LASSO coefficient profiles of the eleven factors. 10-fold cross-validation was used to plot the dotted vertical line at the chosen value.

# SUPPLEMENTARY MATERIAL 3 - Figure. 2


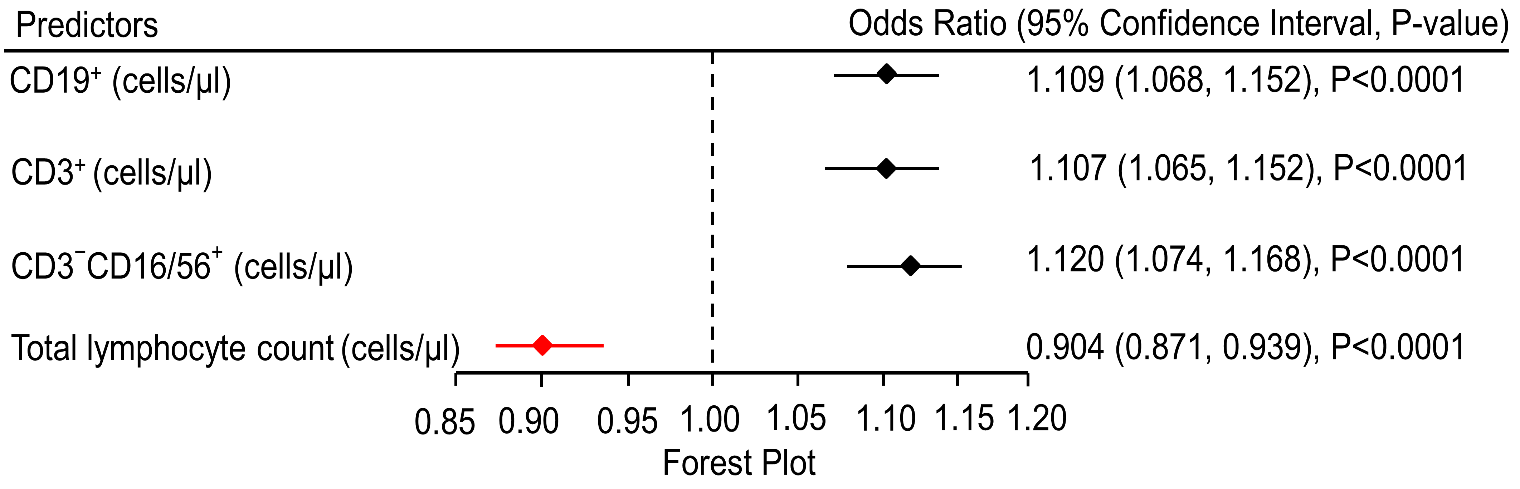


**Figure. 2** The forest plot displayed the multivariable logistic regression model for CD19^+^ B cell counts, CD3^+^ T cell counts, CD3^-^CD16/56^+^ NK cell counts, and total lymphocyte counts.

# SUPPLEMENTARY MATERIAL 3 - Figure. 3

**
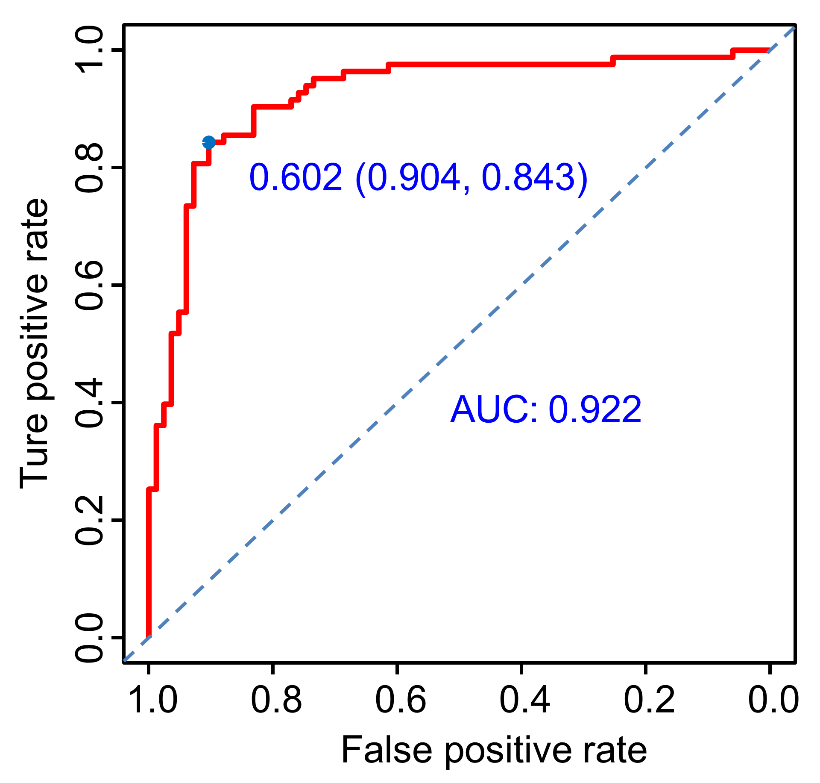
**

**Figure. 3** ROC curve of differential diagnosis nomogram showing an AUC of 0.922, the optimum cut-off value of this nomogram was 0.602, the sensitivity of 0.904, and specificity of 0.843.

# SUPPLEMENTARY MATERIAL 3 - Figure. 3

**
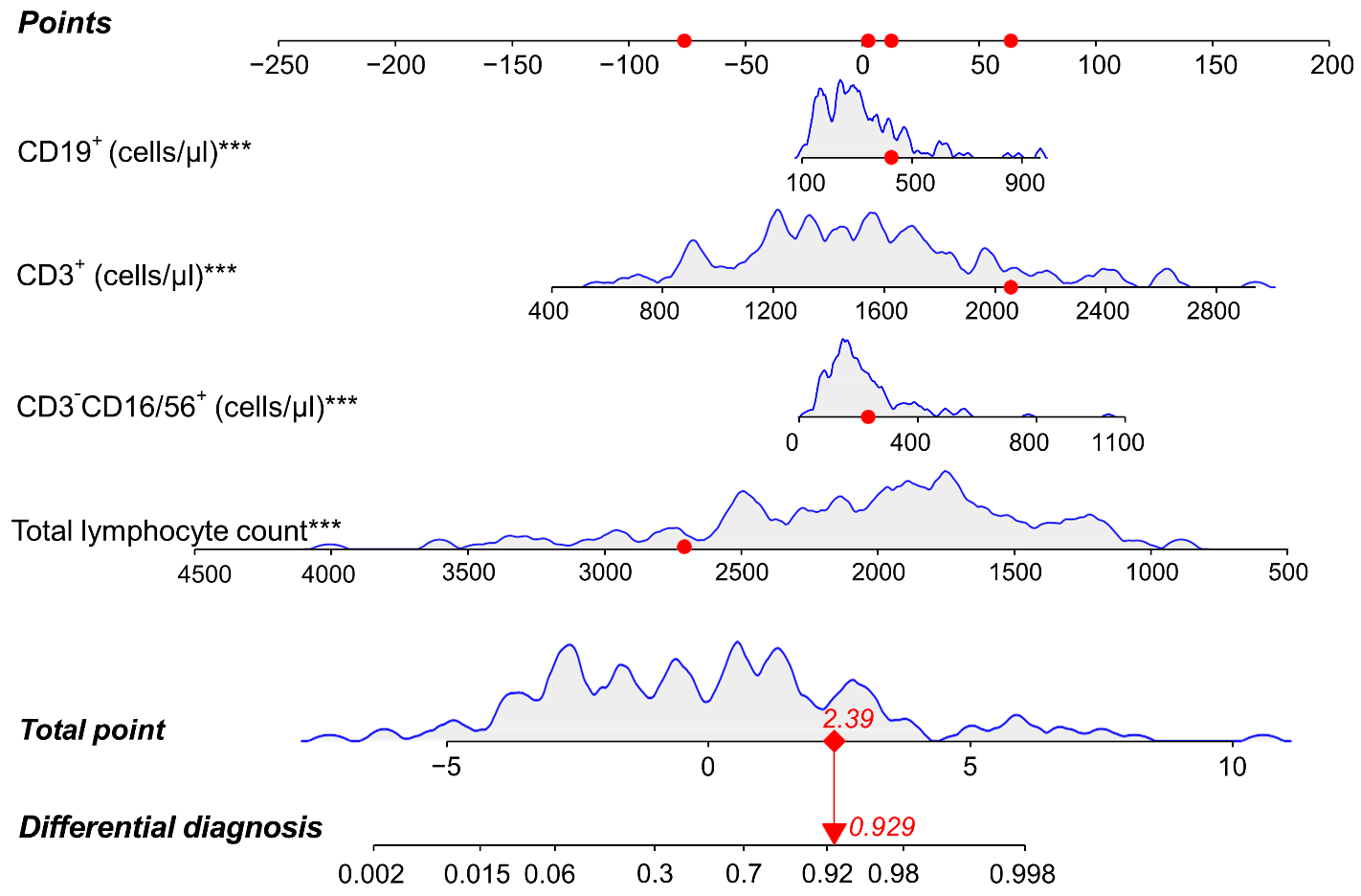
**

**Figure. 4** A nomogram for differential diagnosis in participants with BD and MDD. The study cohort developed a nomogram that included CD19^+^ B cell counts, CD3^+^ T cell counts, CD3^-^CD16/56^+^ NK cell counts, and total lymphocyte count. First, the predictors should always be located on the corresponding axes. Then the corresponding point for each predictor is determined by drawing a line upward, and finally, by adding the total point of the four predictors, the corresponding diagnostic probability is obtained as the predicted value of BD. For example, the risk of BD in one participant, who has a CD19^+^ B cell counts, CD3^+^ T cell counts, CD3^-^CD16/56^+^ NK cell counts, and total lymphocyte counts of 424.8, 2056.6, 233.4, and 2706 (cells/1μl), respectively, could be calculated to be 92.9%.

# SUPPLEMENTARY MATERIAL 3 - Figure. 5


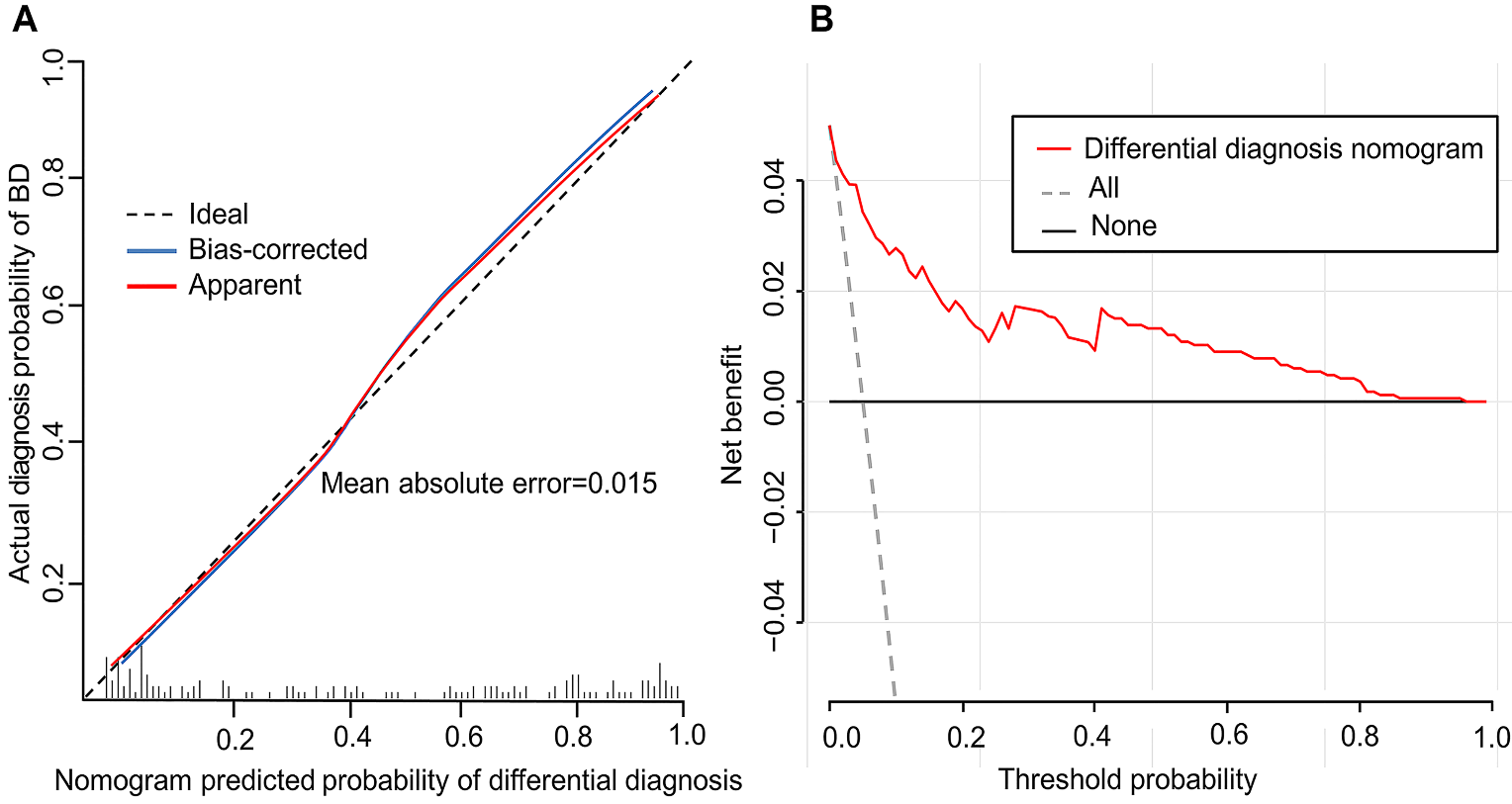


**Figure.** **5** The calibration plot and DCA curve of differential diagnosis nomogram. **(A)** The calibration plot of differential diagnosis nomogram with a 1000 repetition bootstrap (mean absolute error of 0.015). The nomogram predicted probability of differential diagnosis was shown on the x-axis. The actual diagnosis probability of BD was shown on the y-axis. The black dotted line denoted the location of the ideal nomogram, which has identical predicted and actual probabilities. The red solid line represented the apparent accuracy of the nomogram without overfitting correction, with closer to the ideal curve indicating good predicted performance. The blue solid line represented the bootstrap-corrected nomogram. **(B)** DCA demonstrated the net benefit of using the differential diagnosis nomogram to predict BD and MDD diagnosis. The net benefit was shown on the y-axis. The differential diagnosis nomogram was represented by the red solid line. The grey dotted line represented the assumption that all patients could be identified. The black solid line parallel to the x-axis represented the assumption that no patients could be identified. The DCA curve showed that using a nomogram to diagnose BD or MDD can provide significant benefit if the patient or doctor is within a threshold probability of greater than 5%.
