# Supplementary figures and images for: Development and validation of a nomogram based on lymphocyte subsets to distinguish bipolar depression from major depressive disorder
Source: Front Psychiatry. 2022 Oct 6;13:1017888. doi: 10.3389/fpsyt.2022.1017888 (PMC9583168; doi:10.3389/fpsyt.2022.1017888)

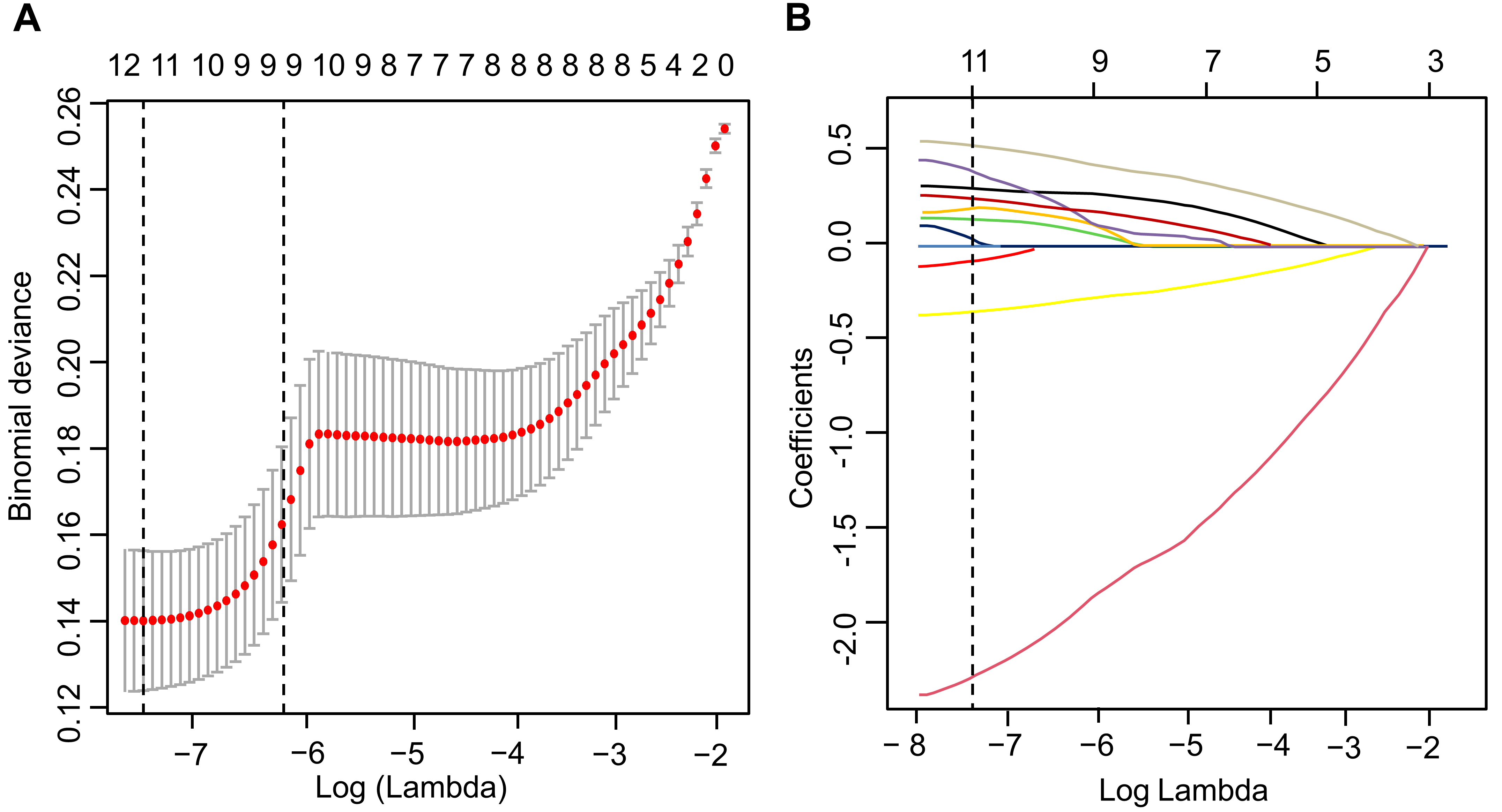

Supplement: Supplementary file 4 [file Presentation_1.ZIP › Supplementary Material Presentation/Frgure.1AB.tif]

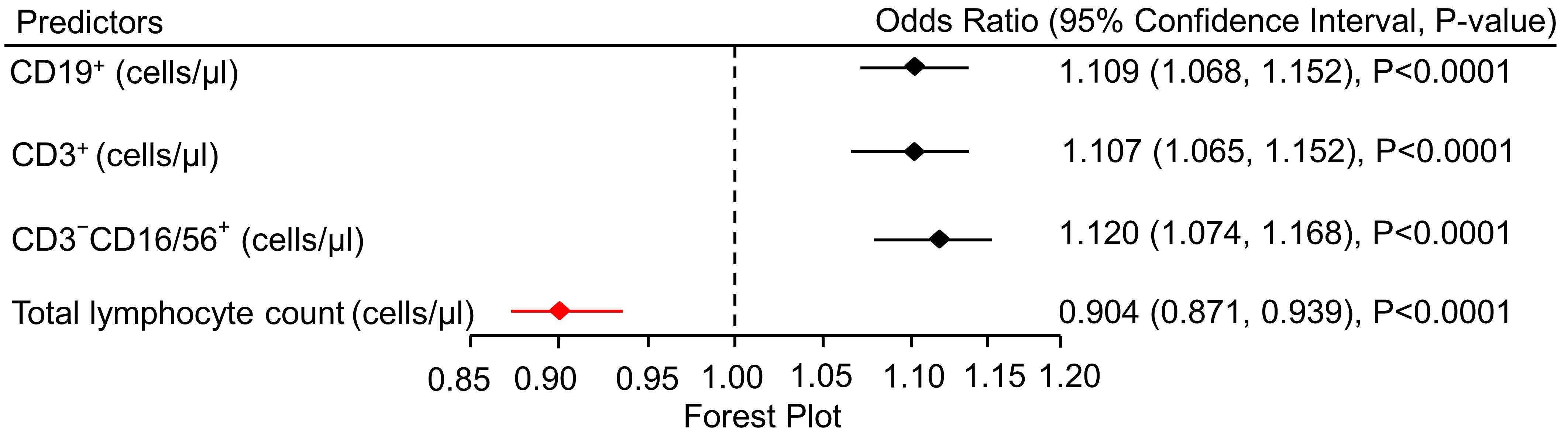

Supplement: Supplementary file 4 [file Presentation_1.ZIP › Supplementary Material Presentation/Frgure.2.tif]

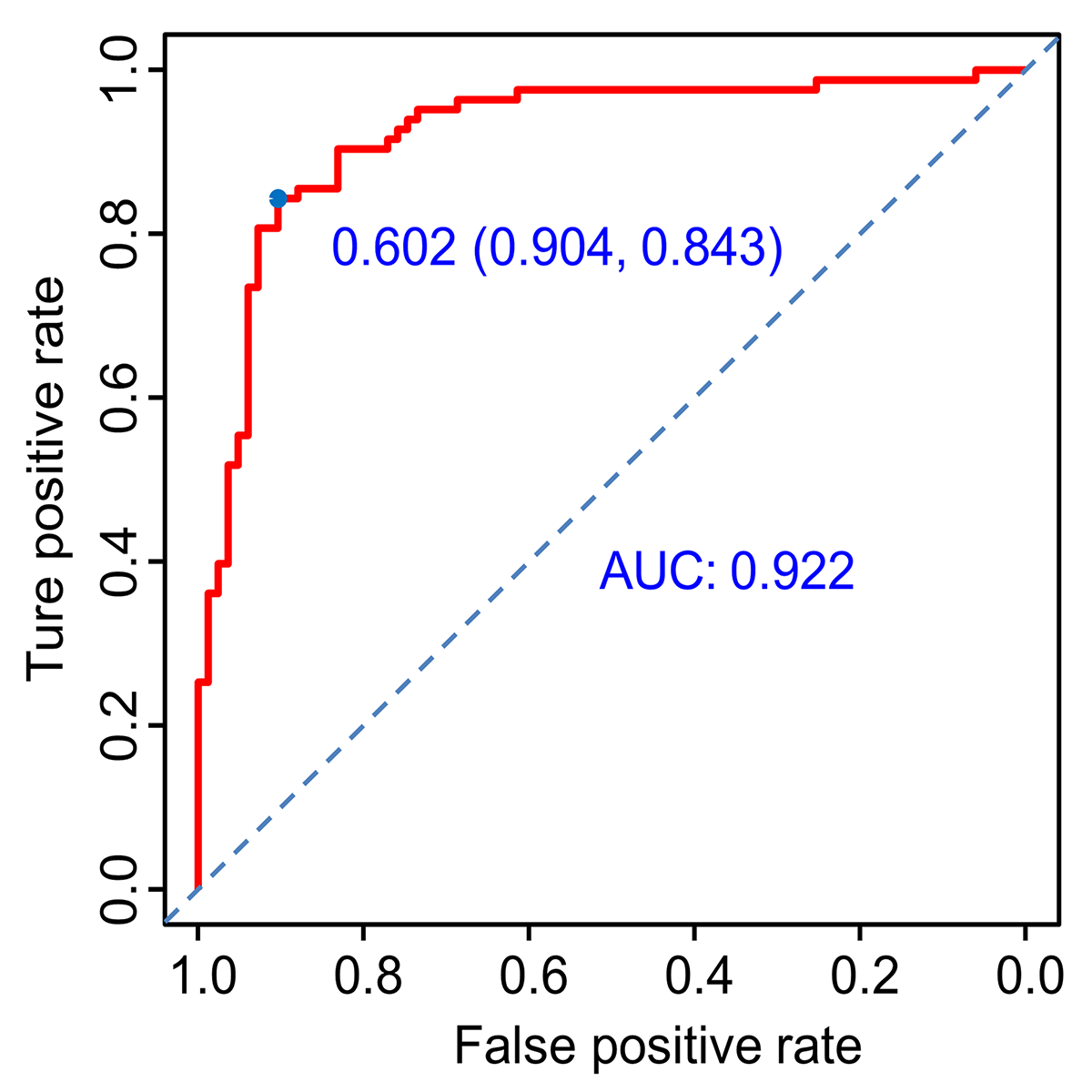

Supplement: Supplementary file 4 [file Presentation_1.ZIP › Supplementary Material Presentation/Frgure.3.tif]

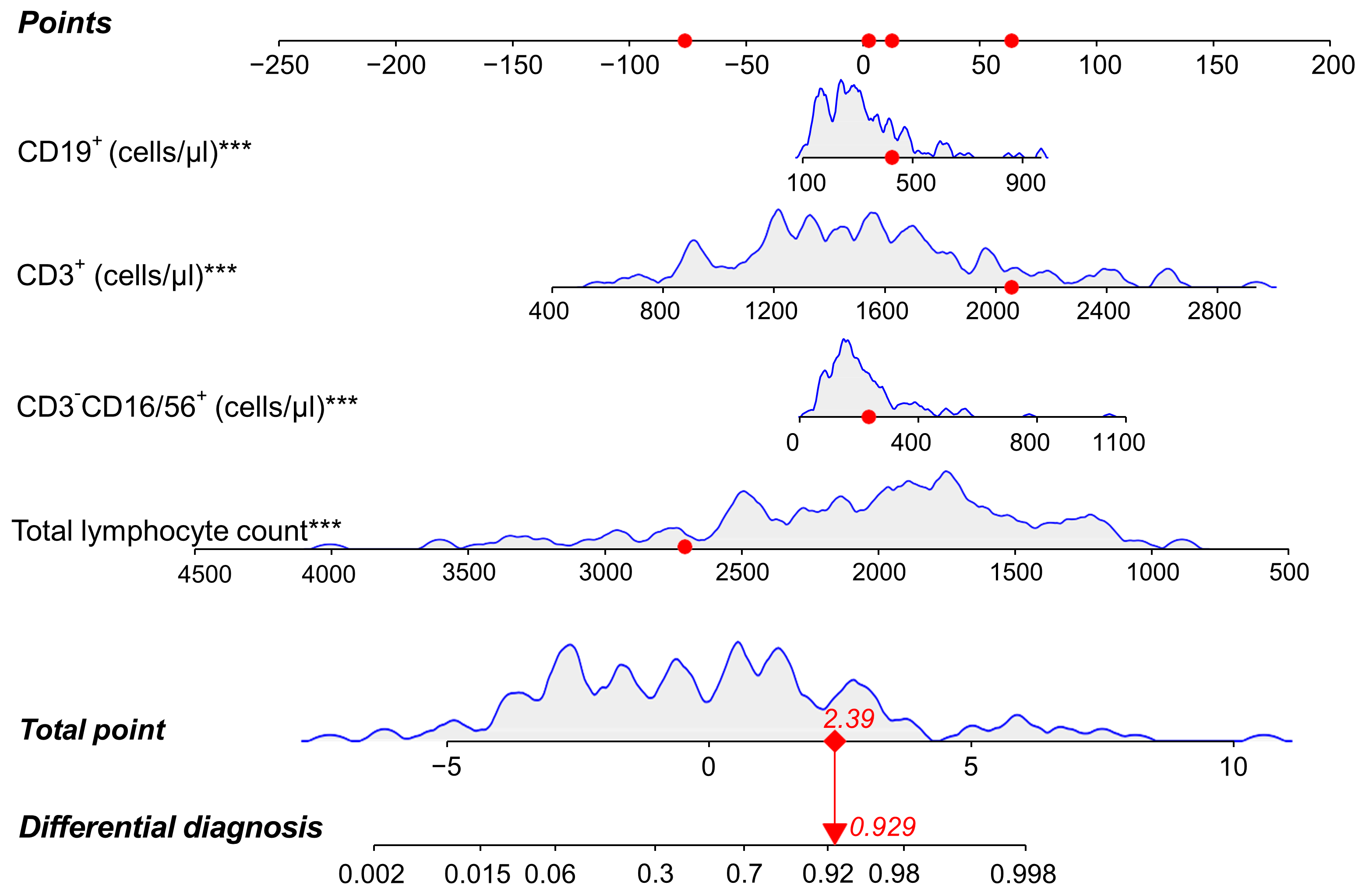

Supplement: Supplementary file 4 [file Presentation_1.ZIP › Supplementary Material Presentation/Frgure.4.tif]

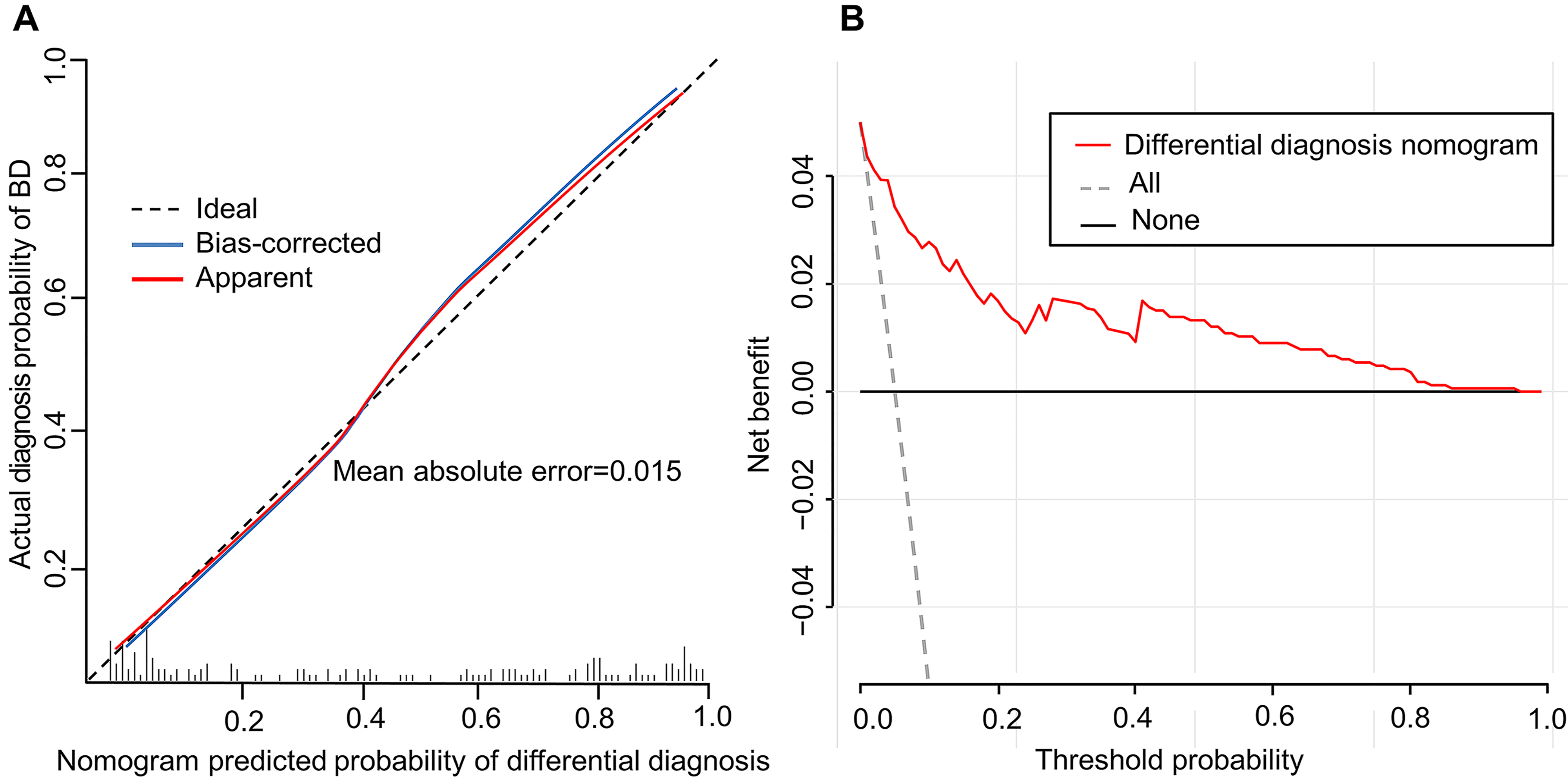

Supplement: Supplementary file 4 [file Presentation_1.ZIP › Supplementary Material Presentation/Frgure.5 AB.tif]
